# Ketamine Use in Self-Described Therapeutic Contexts: A Thematic Analysis of Reddit Posts

**DOI:** 10.3390/bs16040480

**Published:** 2026-03-24

**Authors:** Jared Kendrick, Ghonwa Ahmad, Audrey Wood, Samuel Stumo, Aarav Sehgal, Douglas B. Matthews, Pravesh Sharma

**Affiliations:** 1 Department of Psychology, University of Wisconsin—Eau Claire, 124 Garfield Ave., Eau Claire, WI 54701, USA; kendrijn7239@uwec.edu (J.K.); woodat0836@uwec.edu (A.W.); stumosm8453@uwec.edu (S.S.); matthedb@uwec.edu (D.B.M.); 2 Faculty of Medicine, Yarmouk University, Shafiq Irshidat St., Irbid 21163, Jordan; ghonwa22@gmail.com; 3 Sachem High School East, 177 Granny Rd., Farmingville, NY 11738, USA; sehgal.aarav@gmail.com; 4 Department of Psychiatry and Psychology, Mayo Clinic Health System, 1221 Whipple Street, Eau Claire, WI 54703, USA

**Keywords:** ketamine, reddit, thematic analysis, user experience

## Abstract

The use of ketamine for the management of neuropsychiatric conditions outside clinical settings has rapidly expanded, creating a critical need to understand diverse individual experiences. We conducted a qualitative content analysis of posts from the r/TherapeuticKetamine subreddit. From 3302 threads, the 500 highest-engagement threads (12,852 comments) were analyzed by independent coders across six domains: perceived positive effects, adverse effects, reasons for use, route of administration, polydrug use, and dose amounts. Mood-related concerns were the primary reason for use (53%). Users reported positive effects, most often improvements in emotional well-being (65%). Adverse effects were predominantly psychological or mood-related (56%). A total of 70% of reported doses exceeded 149 mg, suggesting a trend toward higher dose use. Intravenous administration (40%) and sublingual troches (23%) were the most frequently reported routes. Concurrent use of prescribed psychotropics, cannabis, and psychedelics was also reported. This analysis identified substantial heterogeneity in individual-reported experiences. Frequent high-dose use, dose escalation, and polydrug exposure underscores the importance of clinical monitoring and attention to addiction potential and drug–drug interactions. The findings should be interpreted with caution, as follow-up and clinical verification were not possible; however, the data provide an unfiltered view of individual experiences in relation to ketamine use outside the clinical setting.

## 1. Introduction

Ketamine is a fast-acting general anesthetic that was first synthesized in 1962 by Calvin L. Stevens (Dorandeu, 2013; Morgan et al., 2012; Simonini et al., 2022; Zhou et al., 2023). Following its synthesis, ketamine was authorized for human clinical investigation in 1964 (Morgan et al., 2012) and subsequently received the United States (US) Food and Drug Administration (FDA) approval for use as a general anesthetic, after which it became increasingly incorporated into surgical anesthesia and perioperative analgesic practices (Gao et al., 2016; Simonini et al., 2022). The initial formulation of ketamine was developed as a racemic preparation consisting of an equimolar (1:1) mixture of the S-(+)- and R-(−)-ketamine enantiomers, a distinction that has gained relevance due to differences in each enantiomer’s pharmacologic and clinical profiles (Ulrich Zeilhofer et al., 1992). The term ketamine is often used in both clinical and public discourse, referring variably to racemic ketamine (a 1:1 mixture of the R- and S-enantiomers approved by the FDA as an anesthetic) or to esketamine (Quintero et al., 2025; Trimmel et al., 2025), the purified S-enantiomer approved in 2019 as an intranasal formulation for treatment-resistant depression under a Risk Evaluation and Mitigation Strategy (REMS) (Cristea & Naudet, 2019). This ambiguity is particularly relevant in online discussions, where users frequently refer to “ketamine” without specifying formulation, route of administration, or regulatory status.

The use of ketamine-based treatments has increased substantially in recent years (Na & Kim, 2021; Yang et al., 2025). As of 2024, an estimated 500 to 750 independent ketamine clinics were operating within the US, up from approximately 300 in 2018 (Megli, 2024; Reardon, 2018). While off-label prescribing is legally permissible, route of administration has important implications for risk and clinical oversight (Sanacora et al., 2017; Sayad et al., 2025). When administered intravenously, ketamine has well-documented acute psychoactive and cardiovascular effects, including dissociation and transient increases in blood pressure and heart rate (Ansari et al., 2024; Goddard et al., 2021; Kitch, 2020). Consequently, in anesthesia and hospital-based settings, intravenous ketamine is typically delivered with continuous physiological monitoring and immediate access to emergency support (Jelen et al., 2024; Marland et al., 2013).

A challenge in characterizing real-world ketamine use is the potential for incomplete disclosure within clinical settings (Chi & Chen, 2023; Hartwig et al., 2025). Individuals who use ketamine outside of prescribed frameworks, including those who supplement or substitute prescribed treatment with non-prescribed ketamine, may be reluctant to report such use or related adverse effects to clinicians because of stigma, legal concerns, or fear of treatment discontinuation (Harding et al., 2025; Jilka et al., 2019). As a result, clinician-facing data sources may underestimate the diversity of ketamine use patterns, co-use behaviors, and adverse experiences occurring outside tightly monitored clinical environments (Yang et al., 2025).

Social media platforms provide a complementary lens for examining these phenomena, as they may allow individuals to discuss experiences with substances (ketamine) including non-prescribed use, adverse effects, and self-directed dosing practices in a pseudonymous environment that may reduce barriers to disclosure (Fan et al., 2017; Raza et al., 2023). Prior research has demonstrated that online forums capture forms of substance use, self-medication, and treatment-related concerns that are often underreported in traditional clinical or survey-based studies (Chiauzzi et al., 2013; Hartwig et al., 2025). Examining these discussions may therefore help identify experiences and risks that remain obscured in formal healthcare settings.

Although ketamine is increasingly discussed as a therapeutic intervention in public forums including media (Thornton et al., 2023), its non-clinical, recreational, or improperly supervised use may frequently occur outside standardized dosing, monitoring, and prescribing frameworks (Harding et al., 2025). In such contexts, distinctions between therapeutic use, self-medication, and misuse may be blurred (Harding et al., 2025; Kuntz, 2023). From a substance use and public health perspective, these settings warrant surveillance for emerging risk signals, including dose escalation, polydrug exposure, and adverse psychological effects. Despite its clinical and research utility, ketamine carries recognized risks of misuse, psychological dependence, and bladder and cognitive dysfunctions. These risks are particularly salient with off-label use where standardized monitoring measures are absent (Harding et al., 2025; Ingrosso et al., 2025; Ng et al., 2026).

Accordingly, we analyzed the subreddit r/TherapeuticKetamine, which at the time of data collection included approximately 37,000 subscribers and 600 weekly contributions. The users routinely shared experiences with ketamine use, including dosing protocols, routes of administration, and perceived benefits and challenges. These discussions provided a naturalistic window into individual-reported effects and practices, while potentially reducing biases inherent to traditional surveys and interviews. Confirmation of prescription status was not possible, and the r/TherapeuticKetamine forum may attract non-prescribed and recreational ketamine users seeking peer validation, likely blurring the boundary between therapeutic vs. non-therapeutic use. Reddit is a popular anonymous social networking forum (Antisdel et al., 2025; Guo & Caine, 2025), with approximately 116 million daily active users (Reddit, Inc., 2026). User profiles typically neither provide nor link to information identifying individuals, and usernames are overwhelmingly pseudonyms (Guo & Caine, 2025). Setting up a profile picture and posting a short biography is optional (Hartwig et al., 2025). Subreddits are topic-based forums within Reddit, moderated by volunteer moderators (Goglia & Vega, 2024). Users can subscribe to a subreddit for easy access and participate in subreddits by commenting on existing threads or creating new threads (Goglia & Vega, 2024). Moderators ensure that users are respectful and relevant by reviewing threads, suggesting changes, and removing posts that do not meet community standards (Goglia & Vega, 2024).

The present study sought to characterize the subjective experiences of individuals using ketamine for self-reported therapeutic purposes by analyzing user-generated discussions within the r/TherapeuticKetamine community. Specifically, we aimed to examine users’ reported reasons for use, dosing practices, perceived benefits, adverse effects, and patterns of co-use with other substances (polydrug use). Given the exploratory nature of this study, we did not posit a directional hypothesis. However, we anticipated that user-reported experiences would reflect both perceived therapeutic benefits and adverse effects, that these accounts would differ meaningfully from standard clinical settings, and that there would be heterogeneity in individual experiences.

## 2. Materials and Methods

### 2.1. Overview

The present study employs a qualitative content analysis (Hsieh & Shannon, 2005; Lyhne et al., 2025; Raza et al., 2023) to examine discussions related to ketamine on one of the largest social media platforms, Reddit (reddit.com). Reddit was selected to complement information typically obtained through clinical encounters, which are inherently constrained to individuals who present to medical providers—often those experiencing clinically salient concerns or adverse outcomes (National Institute on Drug Abuse, 2025; Sayad et al., 2025). Anonymous online forums such as Reddit can provide access to a broader and less selectively filtered population of ketamine users, including individuals who use ketamine for self-disclosed therapeutic purposes but may not engage with formal healthcare systems.

This study focuses specifically on the “therapeutic ketamine” subreddit, a topic-centered online community dedicated to discussions of ketamine for self-reported therapeutic use, thereby offering a unique window into naturally occurring, user-driven narratives. The anonymity afforded by Reddit facilitates more candid discourse, enabling users to discuss a wider range of experiences, motivations, perceived benefits, and harms than would likely be disclosed in identifiable or clinical settings, particularly given the normative, legal, and professional risks associated with ketamine use (Center for Drug Evaluation and Research, 2023). However, with the anonymity offered by Reddit, such platforms may enable fabrication, exaggeration, and role-play experience with ketamine (Chaves et al., 2023). Therefore, the data should be interpreted with caution.

### 2.2. Data Extraction

**Figure 1:**
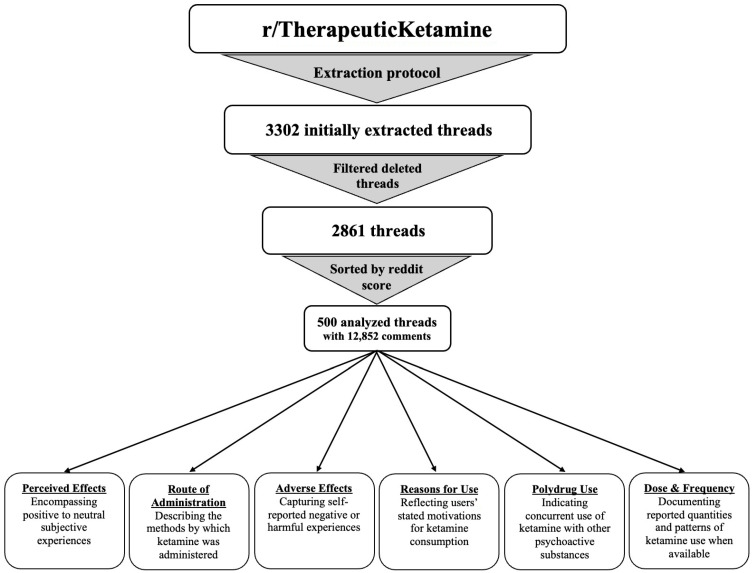
A flowchart of the data extraction and filtering process. The figure describes a flow diagram depicting Reddit thread selection and filtering, from initial extraction to final analytic sample (500 threads; 12,852 comments).

Reddit thread links and metadata from 3 July 2024 to 3 July 2025 were extracted using the Arctic Shift API download tool (https://arctic-shift.photon-reddit.com/download-tool (accessed on 15 December 2025)) (ArthurHeitmann, 2023/2026). Initially, 3302 threads from the r/therapeuticketamine reddit community were downloaded. An initial filtering protocol was followed that removed any threads that were deleted by users or community moderators. A total of 441 threads from r/therapeuticketamine were removed following these criteria. The remaining threads were sorted by descending “reddit score”, defined as the sum of upvotes (+1) and downvotes (−1). From these remaining threads, the 500 threads with the highest score from the subreddit were analyzed. This analysis included 12,852 comments that were contained within the threads (Figure 1).

### 2.3. Analysis

A random sample of 20 threads from the selected subreddit was analyzed to identify salient themes and inform code development. Substantial relevant data were contained within the associated comment sections, which were therefore included in the analysis. Across sampled threads, the number of comments ranged from 0 to 280. Given the density and richness of these user-generated responses, comments were incorporated into the sentiment analysis (Villanueva-Miranda et al., 2025) alongside original posts, with each comment being analyzed with the same detail afforded to the original post. This screening analysis involved reviewing language used by commenters with the goal of identifying specific analytic domains that were most common and relevant to users’ experience while using ketamine. This initial screening process yielded the following analytic domains associated with ketamine use for self-reported therapeutic purpose: (1) Perceived effects, encompassing positive to neutral subjective experiences; (2) Adverse effects, capturing self-reported negative or harmful experiences; (3) Reasons for use, reflecting users’ stated motivations for the use; (4) Route of administration, describing the methods by which ketamine was self-used; (5) Polydrug use, indicating concurrent use of ketamine with other substances or drugs; and (6) Dose and frequency, documenting reported quantities, when available.

The top 500 threads were subsequently analyzed by four independent coders. Coders received training from JK (graduate student) and GA (graduated medical student), who have prior experience conducting similar analysis, including in previously published work (Hartwig et al., 2025). During initial thread coding, coders were instructed to track multi-entry users and count repeated reports by a user as a single instance. When a user appeared multiple times, their reports were aggregated, with repeated reports only counting once. Comments left by the thread’s original poster (OP) are tagged with an “OP” tag in any comments left by them in their own post. Other commenters required careful tracking by coders while reading each thread. Following initial open coding of relevant language, the coders met to resolve discrepancies and refined the coding framework. To assess inter-coder consistency, a random subsample of 25 posts was independently cross-coded and compared across coders. After consensus was achieved, the research team convened to identify higher-order themes, into which semantically related user language was grouped. When disagreements could not be resolved, the senior author (PS), an expert in qualitative research methodology, was consulted. Since coding and analysis was consensus based, inter-rater reliability statistics were not calculated.

Themes were retained if they (a) appeared more than ten times across the dataset, (b) captured conceptually distinct phenomena, or (c) did not readily align with existing thematic categories. This thematic identification process was conducted separately for each analytic domain. Once themes were finalized, coders reviewed the code sheets to quantify the frequency of specific language units and aggregate them into thematic statements. The resulting theme statements are presented in the left-hand column of each table, with illustrative examples of representative user language, along with their corresponding frequencies, displayed in the right-hand column. 

## 3. Results

### 3.1. Positive Effects

There were 2726 written occurrences of positive effects over 500 posts (Table 1). These occurrences contained a wide range of observed effects for those using ketamine in a self-reported therapeutic context. The most common effect noted in the posts were improvements in emotional and psychological well-being, with 1760 (65%) recorded positive effects falling under this category. The commenters used a variety of terminology when referring to improvements in psychological well-being; therefore, the coders were instructed to use the terminology that was most commonly used by the commenters. For example, if commenters mentioned improvements in “depression,” “anxiety,” and other psychiatric conditions, these were categorized as improved psychological illness. If they mentioned improvements with more general terms, such as “mood,” “happiness,” and related concerns, it was categorized as improved mood. Any other reasons related to emotional and psychological wellness were placed in the broader category of improved mental health. The second most reported positive effect was altered experiences (dissociation, hallucinations, or feeling high), with 760 (28%) of the recorded positive effects falling under this category. Altered experiences—such as dissociation or hallucinations—were coded as positive effects only when participants clearly described them as desirable or helpful for their perceived therapeutic process. Psychedelic experience was coded for any reports that included terminology such as, “psychedelic” and “trip”. This classification was based on the users’ own interpretation of the experience, rather than on clinical definitions, in which such experiences may be considered adverse effects. Therefore, this approach reflects the nuanced experiences as reported by users. Any disagreements about how to classify the valence of an experience were discussed and resolved through consensus. Other observations included reported cognitive (improvements in memory and cognition), physiological or physical improvements (improvement in physiological condition, feeling energized, sexual effects), although these were less frequently reported. Reports of positive effects captured both acute and longer-term experiences; however, time course was not systematically coded as a distinct variable, and temporal categorization of individual effects should be interpreted with caution.

### 3.2. Adverse Effects

Psychological and mood-related effects were the most frequently described adverse effects, accounting for 538 reports (56%). This category encompassed psychological symptoms such as fear and emotional distress, as well as reported negative changes in mood (Table 2). Addictive effects represented a clinically distinct pattern of adverse reporting, with 87 mentions—approximately 14% of all psychological effect references. Reported indicators included tolerance leading to dose escalation (60 of 87 reports), withdrawal (19 of 87 reports), and cravings (8 of 87 reports). Other frequently reported categories included neurological, sensory, and gastrointestinal effects. Less frequently reported adverse effects included sleep-related problems, musculoskeletal, urological, cardiovascular, respiratory, sexual, and exocrine/glandular effects.

### 3.3. Reasons for Use

There were 1978 written occurrences of reasons for use across 500 posts (Table 3). Because this is a therapeutic ketamine group, we aimed to obtain more granular information regarding users’ motivations and experiences. Therefore, for reasons for use, we coded the comments to the lowest possible level of specificity and retained the exact terminology used by commenters. The most common reason for use in the posts was mood-related concerns, with 1046 (53%) of recorded reasons falling under this category, including depressive symptoms, anhedonia, and grief. The second most reported reason for use was trauma-related concerns, with 235 (12%) reports describing use for post-traumatic stress disorder (PTSD) and other unspecified trauma. Other observed reasons for use included anxiety-related concerns, substance use reduction, and neurodevelopmental concerns, although these were less frequently mentioned. Substance use reduction was counted as a reason for use for any users who reported using ketamine to assist in reducing their use of other substances such as alcohol, marijuana, or any other substance noted in the discussion.

### 3.4. Routes of Administration

A total of 601 drug administration events were recorded (Table 4). Injections constituted the most common method of administration, accounting for nearly half of all events (~49%). Among injection routes, intravenous administration was most frequently reported (n = 238), followed by intramuscular injection (n = 54). Sublingual routes accounted for approximately 35% of administration events, including troches (n = 140), rapid-dissolving tablets (n = 52), and other sublingual methods (n = 17). Intranasal administration was reported in 14% of events, with nasal sprays representing the predominant intranasal method (n = 66). Oral (n = 13) and rectal (n = 3) routes were infrequently reported and together accounted for less than 3% of all administration events.

### 3.5. Polydrug Use

Collectively, the co-reported agents represented multiple pharmacologic classes (Table 5). The most described co-use involved ketamine-induced symptom management or adjunctive medications, particularly antiemetics and sedating agents. Ondansetron was frequently mentioned in this context, alongside antihistaminic/sedating agents (e.g., diphenhydramine) and occasional reports of other supportive medications (e.g., promethazine), as well as autonomic or anxiety-related agents (e.g., propranolol) and herbal supplements (e.g., valerian root).

Concomitant antidepressant therapy was also widely reported. Mentions included antidepressants (SSRIs/SNRIs, including venlafaxine and fluoxetine) as well as other antidepressant classes such as tricyclic agents and newer or atypical antidepressants (e.g., bupropion-containing regimens and dextromethorphan–bupropion). Stimulant medications co-use included amphetamine-based formulations and methylphenidate. Co-use with anxiolytic or sedative–hypnotic agents was also described, particularly benzodiazepines (e.g., clonazepam, lorazepam, midazolam, diazepam, and alprazolam). Other centrally acting agents that were mentioned but did not fit neatly into a single category included duloxetine, atomoxetine, magnesium supplementation, and related adjuncts.

Less frequently, users reported concomitant antipsychotic medications, including partial dopamine agonists and sedating antipsychotics (e.g., aripiprazole, brexpiprazole, quetiapine, and cariprazine). Metabolic or weight-related agents were rarely noted, including GLP-1-based medications.

Finally, a subset of reports described non-prescribed psychoactive substance use in temporal proximity to ketamine, most commonly cannabis/THC products and classic psychedelics (e.g., psilocybin-containing mushrooms), with occasional mentions of other substances such as MDMA and nicotine through vaping.

These co-use patterns are particularly concerning in the context of off-label, unsupervised ketamine use, which lacks FDA approval for psychiatric indications and standardized safety monitoring requirements.

### 3.6. Doses

Dose information was reported in 193 posts across the dataset (Table 6). Only explicitly stated and numeric dose reports were accounted for, with vague language such as “a lot”, “double my dose”, and other imprecise accounts being left out of analysis. Substantial variability in dosing practices was observed, and reported doses were grouped into five categories based on total milligram amount per session. The most frequently reported category was very high doses (300–749 mg), accounting for 69 reports (35%), followed by high doses (150–299 mg) with 60 reports (31%). Moderate doses (50–149 mg) were reported in 46 posts (24%), whereas low doses (<50 mg) were infrequently mentioned (11 reports; 6%). A small number of posts described extreme doses (≥750 mg) (7 reports; 4%). Overall, 70% of reported doses exceeded 149 mg, indicating that the majority of users who disclosed dose information described use at high to extreme dose ranges.

## 4. Discussion

This study presents the results of content analysis of posts from the r/TherapeuticKetamine subreddit. Our team extracted 3302 threads posted over a one-year period and analyzed the 500 highest-engagement threads comprising 12,852 comments. Six overarching domains were identified and analyzed by four independent coders: perceived positive effects, adverse effects, reasons for use, route of administration, polydrug use, and dose amounts. Mood-related concerns were the most reported reason for ketamine use (53%). Users frequently reported positive effects, most often improvements in emotional well-being (65%). Adverse effects were predominantly psychological (fear, emotional distress, and addictive effects, as well as reported negative changes in mood) (56%). Notably, 70% of reported doses exceeded 149 mg, suggesting a trend toward higher-dose use. Intravenous administration (40%) and sublingual troches (23%) were the most frequently reported routes. Concurrent and varied use of psychotropics, cannabis, and psychedelics was also reported.

Our analysis indicated that ketamine use was most frequently motivated by mood and emotional well-being concerns, which also constituted the most commonly reported perceived benefits, aligning with the predominance of mood-related and trauma-associated use patterns in this dataset. This alignment is also consistent with existing clinical and translational research emphasizing ketamine’s rapid effects on mood symptoms (Kang et al., 2022), though the findings within this dataset reflect user-reported experiences rather than clinically verified outcomes.

Frequent reports of altered experiences in our analysis, including dissociation and perceptual changes as a positive effect of self-reported therapeutic use of ketamine, warrant careful clinical consideration. In conventional clinical contexts, such experiences may typically be classified as adverse effects or side effects. In contrast, users in this sample often described these experiences as meaningful or subjectively beneficial (Ballard & Zarate, 2020; Marguilho et al., 2023). These findings highlight substantial heterogeneity in how ketamine’s psychoactive effects are experienced and interpreted, underscoring the need for careful patient education and monitoring in clinical practice.

Across posts, users reported adverse effects spanning psychological/perceptual, neurological, gastrointestinal, urological, cardiovascular, and respiratory domains. This breadth is consistent with ketamine’s known dissociative and systemic physiological effects and reflects how individuals describe and prioritize harm in unsupervised settings, including at-home use, rather than within tightly monitored trial environments (J.-H. Li et al., 2011; Marguilho et al., 2023; Morgan et al., 2012).

Psychological and perceptual effects particularly dissociation and perceptual disturbance (commonly reported as “spaced out,” “detachment” and distorted perceptions) were mentioned as adverse experiences by many and align with established clinical descriptions of ketamine’s pharmacological profile (Dutton et al., 2023; L. Li & Vlisides, 2016). In clinical trials, these effects are commonly anticipated and assessed within short post-dose monitoring windows (Bayes et al., 2022; Daly et al., 2018). Our findings extend this literature by showing that users often describe these experiences in functional terms (e.g., distress, disruption, lingering cognitive or sensory discomfort), which may be more salient when administration occurs outside supervised clinical settings.

Physiological symptoms such as palpitations, blood pressure concerns, or breathing-related discomfort were often framed by users as discrete, sporadic events rather than continuously monitored parameters. While ketamine generally has limited depressant effects on central respiratory drive (Coles et al., 2023; Eikermann et al., 2012) when appropriately administered, variability in dosing, route, and monitoring context may influence how these symptoms are experienced and interpreted by patients.

Gastrointestinal symptoms, particularly nausea, were commonly reported and are consistent with prior perioperative and therapeutic ketamine research (Seo et al., 2025). Although urological symptoms were less frequently described, their presence warrants clinical attention given known associations between cumulative ketamine exposure and lower urinary tract pathology (Chiappini et al., 2025; Wood et al., 2011). Importantly, these reports should not be interpreted as indicating high incidence under medical dosing but underscore the need for clinician awareness, early symptom assessment, and monitoring in unsupervised ketamine use.

The frequent reporting of polydrug use in this dataset suggests that ketamine is commonly used within a broader medication context that includes ongoing prescription psychotropics, symptom management agents, and, for some individuals, non-prescribed psychoactive substances. Many users appeared to employ concomitant medications to mitigate physiological adverse effects commonly associated with ketamine, particularly nausea, vomiting, dizziness, and sedation (Elvir-Lazo et al., 2020; Reddy et al., 2024). This interpretation is supported by mentions of antiemetics (e.g., ondansetron) and sedating or antihistaminic agents (e.g., diphenhydramine and promethazine), which are consistent with symptom management strategies observed in both clinical and informal treatment contexts (Elvir-Lazo et al., 2020; Fiaschetti & Martin-Long, 2026).

Given that mood-related concerns were the most cited reason for ketamine use, the frequent reporting of concurrent antidepressant therapy including SSRIs, SNRIs, and other antidepressant classes is unsurprising. However, the co-use of ketamine with antidepressants, stimulants, benzodiazepines, and other centrally acting agents introduces the potential for pharmacodynamic interactions, particularly with respect to sedation, dissociation, cardiovascular effects, and cognitive impairment (Sayad et al., 2025). The additional appearance of non-prescribed psychoactive substances, most commonly cannabis and classic psychedelics, further highlights co-substance use pattern in users who use ketamine in unmonitored settings (Lake & Lucas, 2025). Overall, these findings suggest that ketamine use in unregulated settings often occurs alongside concurrent prescription medications and, in some cases, non-prescribed substance use, underscoring the complexity of treatment contexts and potential implications for safety, tolerability, and treatment response.

The dose of ketamine used for self-reported therapeutic use was inconsistently reported. Only 193 observations were found. The distribution of reported ketamine doses in this dataset was markedly skewed toward higher absolute milligram amounts, with 70% of dose reports exceeding 149 mg per session. No information was available regarding dosing duration, infusion rate, route-specific bioavailability, clinical supervision, or cumulative exposure, all of which are critical determinants of pharmacologic intensity and risk. We found that ketamine dosing noted in the discussion differed markedly from standard medical practice. Reported doses reflect absolute milligram values rather than weight-based dosing (mg/kg), limiting assessment of individual pharmacologic exposure. Identical reported doses may therefore correspond to substantially different physiological effects across users. In contrast, ketamine for mood disorders in clinical research and practice is typically administered at substantially lower, weight-based doses (e.g., ~0.5 mg/kg IV, or approximately 30–50 mg per session in adults) (Yavi et al., 2022). The divergence between these established therapeutic dosing frameworks and the higher doses commonly reported in online forums highlights a gap between evidence-based practice and unsupervised use patterns.

Although the reported dose ranges partially overlap with cumulative milligram amounts described in select inpatient “burst” ketamine protocols for refractory pain—where several hundred milligrams may be administered over multiple days under continuous medical monitoring (Quibell et al., 2011; Zia et al., 2025)—such overlap should be interpreted with caution. Burst protocols are tightly controlled, indication-specific, and not comparable to the self-directed or unsupervised use. The predominance of higher reported doses is nevertheless concerning given the well-established dose- and exposure-dependent risks associated with ketamine, particularly urological toxicity. Ketamine-induced cystitis severity increases with both higher per-session dosing and repeated use (Anderson et al., 2022). While some users referenced dissociative experiences, such as the “K-hole,” (Muetzelfeldt et al., 2008; Schep et al., 2023), the absence of verifiable context precludes inference regarding intent, frequency, or escalation patterns.

Non-medical ketamine use has increased markedly over the past decade (Harding et al., 2025; Liu et al., 2016). Part of this growth is likely tied to the availability of oral formulations for at-home use which have opened new pathways for misuse and diversion (Palamar et al., 2025). Ketamine use is no longer confined to parties, but it has transitioned into private, unsupervised settings with little to no oversight (Liu et al., 2016; Palamar et al., 2025). Data from the US reflect this shift too: illicit ketamine seizures increased by 349.1% between 2017 and 2022, with the seized volume rising by 1116% in the same period (Harding et al., 2025). Poison center reports of ketamine-related exposures similarly doubled between 2019 and 2023, the highest annual figure ever recorded (Palamar et al., 2025). Ketamine’s addictive potential involves multiple dimensions: psychological reinforcement through dissociative effects, physical complications such as urological problems and abdominal pain, cognitive and neurological changes, and withdrawal symptoms that include cravings, low mood, insomnia, fatigue, tremors, sweating, and palpitations (Harding et al., 2025; Liu et al., 2016). In our sample, addictive effects were reported but less often than psychological effects, possibly because users tend to describe what are, in effect, dependence symptoms in psychological rather than addiction-related terms (Ingrosso et al., 2025; Jansen, 1990, 2000).

This use of ketamine outside the clinical setting raises significant safety concerns for users. Without monitoring by a trained professional, users may be at risk of infection from sharing needles, overdose, and cardiovascular complications such as arrhythmias (Y. Li et al., 2012; Mindwell, 2026; Rap, 2024; Release, 2013). These adverse effects, compounded with ketamine’s dissociative properties, may impair a user’s ability to recognize or respond to adverse reactions in a timely manner. Our findings further reinforce this as many users in our analysis interpreted dissociation, hallucinations and altered perceptions as ‘positive’ experiences, which is particularly concerning as this misattribution may further diminish their ability to recognize potential dangers—another distinction from standard clinical environments. The Reddit data are useful precisely because they capture this gap between how users experience these reactions and how clinicians would categorize them. This insight may help clinicians elicit more accurate information from patients as understanding the language users employ to describe what they experience can guide clinicians in asking more precise questions in clarifying symptom terminology during assessment.

This study has several limitations. The study is based on self-reported content from a social media platform and is therefore subject to selection bias (Chiauzzi et al., 2013; Nesoff et al., 2025), unverifiable clinical status (Chiauzzi et al., 2013; Harrigian & Dredze, 2022), and incomplete reporting of dose, formulation, route of administration, and timing (Chi & Chen, 2023; Chiauzzi et al., 2013; Harrigian & Dredze, 2022; Nesoff et al., 2025; Zhang et al., 2024). Importantly, it is not possible to verify whether reported ketamine use reflects prescribed, clinically supervised treatment versus non-prescribed or informal use, nor to confirm adherence to recommended protocols. Threads were sorted by Reddit score to prioritize high-engagement discussions with rich user participation. However, this strategy may inadvertently favor popular or agreeable content over representative or clinically typical experiences, as higher-scoring posts do not necessarily reflect the breadth of ketamine experiences reported across the platform. The anonymity offered by Reddit increases the potential for users to fabricate or exaggerate ketamine experiences and precludes verification of therapeutic credibility, which compromise the internal validity of the data (Antisdel et al., 2025; Chaves et al., 2023; Chi & Chen, 2023; Chiauzzi et al., 2013). Mentions of co-used substances do not necessarily indicate simultaneous administration, and individual users may contribute multiple posts, limiting independence of observations. Additionally, subreddit-specific demographics and community norms may shape what users choose to disclose, including potential underreporting of stigmatized behaviors. Effects reported by ketamine users also captured both acute and longer-term impacts of their use. The time course of effects was not systematically coded, as users often did not specify this in their posts, leaving uncertainty in whether certain positive or negative effects were experienced acutely or as a later effect (Chi & Chen, 2023; Hsieh & Shannon, 2005; Zhang et al., 2024). These limitations preclude causal inference, prevalence estimation, and longitudinal outcome assessment (Chi & Chen, 2023; Chiauzzi et al., 2013; Nesoff et al., 2025). However, they also allow for the capture of individual-reported experiences, concerns, and use patterns that may fall outside the scope of controlled clinical trials or routine clinical monitoring.

In the future, our team plans to expand this work to Reddit recreational ketamine forums. The reddit community, r/Ketamine, is substantially larger than r/TherapeuticKetamine and enforces fewer restrictions on the content and discussions that are allowed. Applying similar protocols toward this community would allow us to analyze any differences and similarities between therapeutic and recreational ketamine group users (Harding et al., 2025; Lankenau et al., 2008; Palamar et al., 2025). We also aim to expand the use of our content analysis approach to other discussion forums and substances.

## 5. Conclusions

The results of this study corroborate our hypothesis that user-reported experiences reflected both perceived therapeutic benefits and adverse effects, and these accounts differed from experiences that patients report in clinical settings, with large heterogeneity among users. User-reported experiences with ketamine outside of supervised clinical contexts demonstrate that the boundary between perceived therapeutic benefit and harm is often blurred, with experiences frequently spanning multiple, overlapping symptom domains. This complexity stems from the drug’s varied effects, where profound dissociation, euphoria, or spiritual feelings can simultaneously be interpreted by the user as either a valuable psychological insight or a distressing experience.

This analysis demonstrates substantial heterogeneity in individual-reported ketamine experiences, with individuals most commonly seeking benefit for psychiatric symptoms. Within unsupervised ketamine use, individuals report high-dose use and concurrent use of other psychoactive substances. From a clinical perspective, these patterns underscore the importance of careful patient education and proactive monitoring in offsetting potential addiction concerns and drug-drug interactions, particularly when clinicians suspect nontraditional or at-home ketamine use. These findings should be interpreted with caution, as longitudinal follow-up and clinical verification were not possible with social media data. Nevertheless, these reports provide a uniquely unfiltered view of individual experiences that is critical for understanding unsupervised ketamine use beyond formal clinical trials. Together, these findings underscore the utility of social media-based analyses as a digital pharmacovigilance approach for identifying emerging substance use risks at the intersection of therapeutic innovation and informal ketamine use.

## Figures and Tables

**Table 1:** Perceived Positive Effects.

Theme	Specific Positive Effect	Count	Thematic Count
Improvements in emotional or psychological well-being	Improved Mental Health	748	1760
	Improved Psychological Illness	591	
	Improved Mood	271	
	Reduced Substance Use	56	
	Improved interpersonal relationships	52	
	Improved Sleep	42	
Altered experiences (neutral or positive) in tone	Dissociation	328	760
	Hallucinations	254	
	Altered Perception	129	
	Psychedelic Experience	29	
	High	20	
Physiological or physical improvements	Improved Physiological Illness	82	157
	Energized	59	
	Sexual Effects	16	
Cognitive or functional improvements	Cognitive Effects	49	49

**Table 2:** Adverse Effects.

Theme	Adverse Effect	Count	Thematic Count
Psychological- or mood-related	Psychological Effects	440	538
	Altered Mood	88	
	Negative social impact	10	
Neurological or sensory	Neurological Effects	101	176
	Sensory Effects	34	
	Cognitive Effects	34	
	Speech Impairment	7	
Gastrointestinal	Gastrointestinal Effects	116	116
Addictive Effects	Tolerance	60	87
	Withdrawal	19	
	Cravings	8	
Sleep-related	Sleeping Problems	63	63
Musculoskeletal	Musculoskeletal Effects	41	41
Urological	Urological Effects	33	33
Cardiovascular	Cardiovascular Effects	17	17
Respiratory	Respiratory Effects	7	7
Sexual	Sexual Effects (negative)	5	5
Exocrine/glandular	Exocrine Effects	4	4
No adverse effects reported	No effects	30	30
Mentioned but not present in dataset	Pain	0	0
	Nasal Damage	0	

**Table 3:** Reasons for Use.

Theme	Reason for Use	Count	Thematic Count
Mood-related concerns	Depression	997	1046
	Anhedonia	26	
	Grief	23	
Trauma-related concerns	PTSD	215	235
	Trauma (unspecified)	20	
Anxiety-related concerns	Anxiety	193	216
	Panic disorders	18	
	Phobias	5	
Cross-cutting symptoms/general mental health concerns	Suicidal ideation/attempts	102	143
	Nonspecific mental health	36	
	Brain fog	5	
Other medical conditions	Chronic pain	55	109
	Miscellaneous	20	
	CRPS	15	
	Traumatic brain injury	8	
	Migraine	7	
	Chronic fatigue	4	
Neurodevelopmental conditions	ADHD	57	63
	Autism	6	
Reduce substance use	Alcohol	31	57
	Other substances	19	
	Opioids	7	
Induce mental stimulation	To feel high/recreation	30	38
	Spiritual experience/meditation	8	
Obsessive–compulsive-related concerns	OCD	36	36
Bipolar-spectrum concerns	Bipolar	22	22
Sleep-related concerns	Sleeping disorders	6	6
Personality-related concerns	Personality disorders	4	4
Eating-related concerns	Eating disorders	3	3

**Table 4:** Routes of Administration.

Route Category	Specific Route	Count	Thematic Count
Sublingual administration	Troche	140	209
	Rapid dissolve tablet (RDT)	52	
	Sublingual	17	
Injection (parenteral) administration	IV	238	294
	IM	54	
	Unspecified injection	2	
Intranasal administration	Nasal spray	66	82
	Intranasal	16	
Oral administration	Oral (tablet/liquid)	13	13
Rectal administration	Rectal	3	3
Inhalation administration	Inhalation	0	0

**Table 5:** Polydrug Use.

Drug Class	Specific Drug	Count	Class Count
Symptom management/adjunct medications	Antidepressants (unspecified)	13	36
	Zofran	14	
	Benadryl	6	
	Phenergan	1	
	Propranolol	1	
	Valerian	1	
Illegal/non-prescribed psychoactive substances	Weed/Cannabis/THC	12	24
	Psilocybin/Mushrooms	10	
	Molly (MDMA)	1	
	Vaping/Nicotine	1	
SSRI/SNRI and other antidepressants	Effexor	10	25
	SSRI (general)	9	
	Prozac	4	
	Nortriptyline	1	
	Pristiq	1	
Stimulants	Adderall	9	15
	Vyvanse	5	
	Ritalin	1	
Novel antidepressants/atypical agents	Auvelity	8	19
	Wellbutrin	7	
	Bupropion	4	
Benzodiazepines	Clonazepam	6	17
	Ativan	5	
	Midazolam	5	
	Valium	1	
Other/uncategorized psychotropics	Alprazolam	3	12
	Duloxetine	3	
	Magnesium	3	
	Strattera (Atomoxetine)	3	
Antipsychotics	Abilify	2	5
	Rexulti	1	
	Seroquel	1	
	Vraylar	1	
Metabolic/weight-related medications	Semaglutide	1	2
	Tirzepatide	1	

**Table 6:** Reported Doses.

Dose Range	Dose Range (mg)	Total Count
Low dose	<50 mg	11
Moderate dose	50–149 mg	46
High dose	150–299 mg	60
Very high dose	300–749 mg	69
Extreme dose	≥750 mg	7
Total	—	193

## Data Availability

All data were gathered from publicly available reddit archives through the Arctic Shift API download tool.
